# SOCIOCULTURAL DETERMINANTS OF RACIAL HEALTH DISPARITIES ACROSS THE LIFECOURSE

**DOI:** 10.1093/geroni/igz038.2191

**Published:** 2019-11-08

**Authors:** Nancy Chiles Shaffer, Carl V Hill

**Affiliations:** 1 National Institute on Aging, Baltimore, Maryland, United States; 2 NIH/National Institute on Aging, Bethesda, Maryland, United States

## Abstract

Racial health disparities have been observed across the lifecourse. While some studies have attributed these disparities to underlying biomedical differences, a large body of research supports a sociocultural etiology. In this symposium we will demonstrate that observed racial differences in health outcomes are associated with sociocultural factors, and demonstrate effective strategies for assessing race and racial differences in qualitative and quantitative analyses. We will share an overview of health disparities and aging, highlighting the National Institute on Aging’s Health Disparities Research Framework. After this introduction, four speakers will present scientific projects that exemplify the breadth and depth of research assessing health disparities and social determinants of health across the lifecourse. Dr. Brown will present qualitative work on how social determinants are positively addressed through exercise. Dr. Chiles Shaffer will present findings demonstrating the contribution of financial burden and lower education to racial differences in healthy aging. Dr. Noppert will present evidence highlighting the structural influences that underlie the association between education and metabolic health focusing on differences by race and gender. Dr. Thierry will present research on the association between neighborhood characteristics and telomere length with a focus on heterogeneity by race and urban/non-urban residence. Finally, Dr. Hill will provide an overview of the four presentations and facilitate questions and discussion.

